# Modified-mindfulness-based stress reduction as a treatment for cognitive recovery in patients with minor stroke: a randomized controlled pilot study

**DOI:** 10.3389/fneur.2025.1534480

**Published:** 2025-08-26

**Authors:** Sophia G. Girgenti, Isabella Dallasta, Erin Lawrence, Dawn Merbach, Jonathan Z. Simon, Rafael H. Llinas, Neda F. Gould, Elisabeth Breese Marsh

**Affiliations:** ^1^Department of Neurology, Johns Hopkins School of Medicine, Baltimore, MD, United States; ^2^Department of Electrical Engineering, University of Maryland, College Park, MD, United States; ^3^Department of Biology, University of Maryland, College Park, MD, United States; ^4^Department of Psychiatry and Behavioral Sciences, Johns Hopkins School of Medicine, Baltimore, MD, United States

**Keywords:** stroke, recovery, mindfulness, cognitive networks, cognition

## Abstract

**Background:**

Well-developed rehabilitation paradigms exist for post-stroke language and motor impairments. However, no such recovery program has been identified for commonly disabling cognitive deficits in patients following minor stroke. Mindfulness Based Stress Reduction (MBSR) is thought to engage the frontal lobes, improving concentration and attention, and therefore may be an effective option.

**Methods:**

We prospectively enrolled a cohort of patients with subacute minor stroke and randomized them to either an 8-week online modified-MBSR course or online traditional Stroke Support Group (SSG). All patients underwent a battery of cognitive tests and measures of patient reported outcomes (PROs) pre- and post-intervention. ANOVA was used to compare changes in scores over time across both groups, along with a third group of control patients having received neither intervention (*n* = 128).

**Results:**

A total of 30 patients were randomized (*n* = 16 for m-MBSR; *n* = 14 for SSG). The average age of the cohort was 65.9 years. Post-intervention, both groups demonstrated significantly improved T-scores on cognitive tasks, regardless of intervention. Compared to SSG, the m-MBSR group showed a larger degree of improvement in processing speed, executive, and global cognitive function; however, the difference between groups was not statistically significant. Engagement level was not associated with better clinical scores, though was unexpectedly low for both groups.

**Conclusion:**

m-MBSR may modestly improve frontal lobe activity and demonstrates some success in increasing cognitive performance. However, further studies are needed to determine if it is more efficacious in the chronic stage of recovery when more patients are able to fully engage and actively participate.

## Introduction

Stroke is the fifth leading cause of death in the United States (1). With greater than 7.6 million new ischemic strokes globally per year, the estimated cost is over $721 billion US dollars (2). The most common and visible deficits of major strokes, hemiparesis and aphasia, benefit from well-developed evidence-based rehabilitation paradigms (3–6). However, the landscape of recovery is changing as acute treatment with intravenous thrombolytics (7) and mechanical thrombectomy (8) have allowed larger and more disabling strokes to be treated early, decreasing infarct size and resulting in a greater number of “minor” strokes (9). While minor strokes typically spare patients from dependency on long-term care and often allow them to maintain overall independence, they can still be debilitating, resulting in cognitive dysfunction, particularly with respect to executive function, attention, and decision making (10–12). These deficits can be observed on evaluations such as the Montreal Cognitive Assessment (MoCA) (13) and appear consistent across the majority of patients, regardless of stroke size and location (10). Patients with minor stroke are often unable to return to work due to these cognitive deficits, significantly impacting quality of life and making depression and anxiety common (11). Unfortunately, apart from the Stroke Support Groups typically offered at hospitals to give patients and their families an outlet to discuss these difficulties with professionals, treatment and rehabilitation options—specifically for cognitive dysfunction—are significantly lacking.

In search of a novel intervention to address cognitive symptoms associated with minor stroke, mindfulness-based stress reduction (MBSR) is an encouraging alternative. MBSR is a group-based intervention combining meditation, yoga, and body awareness to help individuals cope with stress and better handle life’s challenges. Mindfulness cultivates a purposeful, non-judgmental awareness and acceptance of the present mindset. Originating from the ancient practices of Eastern Buddhist monks, with influences from Western thought (14, 15), this practice is believed to engage the attention networks and frontal lobes, with prior neuroimaging studies involving both expert and novice meditators showing activation of frontal cortex as well as changes in frontal activity while practicing meditation (16–18). These areas are known to play a significant role in executive function, attention, and decision making—all activities hindered in patients with cognitive dysfunction following minor stroke. Mindfulness training in general has been shown to improve health, quality of life, social functioning, and mental health outcomes measured before and after the intervention (19, 20). This makes it an appealing therapeutic option for patients experiencing depression, anxiety, and stress, all common symptoms in survivors of minor stroke (9). MBSR has shown potential benefit in a heterogenous population of patients, with conditions ranging from traumatic brain injury to cancer, depression, and diabetes (21–30).

Improvements in cognitive performance and executive function have also been shown after MBSR intervention in patients with chronic and subacute stroke (between 6 and 36 months post-infarct) (31, 32). Other studies involving patients with stroke have found mindfulness and relaxation techniques to alleviate anxiety (33) and tension (34), and a systematic review found mindfulness-based interventions may provide additional benefit to stroke survivors specifically (35). While it is a concern that impairment from stroke and the long session length may be barriers for this patient population, Jani et al. evaluated stroke survivors’ ability to participate in an abbreviated MBSR study and found potential successful MBSR modifications for stroke survivors (36). Notably, no existing studies have investigated the effects of MBSR on patients with stroke in the early stages of recovery when patients show the most improvement, and more specifically, on the growing population of patients with minor strokes who exhibit specific difficulties with attention and focus.

Patients experiencing minor stroke have tremendous potential to return to their prior level of function and, while the majority improve drastically within the first 6 months post-stroke (37), many fail to successfully re-integrate into society (9). They frequently report difficulties with processing speed, attention, and multi-tasking that impair function and overall quality of life (10). Counseling on eventual improvement of symptoms can be helpful, but, understandably, many desire a treatment that will lead to a faster and fuller recovery. MBSR provides a novel, non-pharmacologic approach that may be effective by targeting areas of abnormality implicated in neuroimaging studies such as the frontoparietal cortex (38). In this study, we explore the effectiveness of MBSR on improving patient-reported outcomes and cognition in patients with recent minor stroke.

## Materials and methods

This was a prospective, randomized, longitudinal pilot study spanning 2 years. Patients admitted for acute stroke to our tertiary referral center were scheduled for a follow-up appointment at the Johns Hopkins Bayview Stroke Intervention Clinic (BaSIC) approximately 6 weeks (±2 weeks) post-infarct. Their data were entered into our Stroke Registry, a HIPAA-compliant, IRB-approved database. Participants were screened before presenting for their first follow-up appointment after hospital discharge and those meeting inclusion criteria were then recruited, consented, randomized, and tested at their clinic visit. After the 8-week intervention, individuals were seen again for repeat evaluation. The study was approved by the Johns Hopkins Institutional Review Board and all participants provided written informed consent at the time of enrollment.

### Inclusion and exclusion criteria

All patients recruited were adults (≥18 years) presenting with their first clinical ischemic stroke. In order to capture small deep lacunes that presented with motor deficits during the acute period, we defined “minor stroke” as a small area of infarction resulting in an admission NIH Stroke Scale (NIHSS) score of 10 or less. This avoided the inclusion of larger cortical lesions localizing to areas that typically cause disrupted cognition, significant hemiparesis, aphasia, or hemispatial neglect. All participants demonstrated evidence on brain imaging of acute ischemic stroke. Strokes were confirmed and infarct volumes were determined using diffusion-weighted magnetic resonance imaging (MRI) and the lesion tool available on the CareStream platform. Lesions were unilateral and supratentorial, without large vessel involvement. Patients had good premorbid baseline function (modified Rankin Scale [mRS] score ≤2).

Patients were excluded if they presented with intracranial hemorrhage, a multifocal stroke involving multiple vascular distributions, or evidence of aphasia or neglect on examination. Those without an MRI or who had no abnormality on diffusion-weighted imaging (consistent with a transient ischemic attack (TIA) or diffusion-negative stroke) were also excluded. Non–native English speakers were excluded, as the testing and interventions were conducted in English, as well as those with a history of significant dementia, prior clinical stroke, neurologic disease, untreated hearing loss, or psychiatric illness. Other exclusion criteria included inability to attend weekly sessions of MBSR or Stroke Support Group (SSG) and inability to return to clinic following intervention. If the patient had another stroke at any point prior to completion of the study, they were excluded from further participation. For those lost-to-follow-up, data was censored at the time they were lost.

### Interventions

At the time of enrollment, participants were randomized to either 8 weeks of an online modified-Mindfulness-Based Stress Reduction (m-MBSR) program or an online conventional Stroke Support Group (SSG). Though traditionally designed as in-person programs, interventions were converted to an online platform due to the COVID pandemic. All participants were provided with an iPad with the Zoom application installed to connect to the assigned weekly meeting. Individuals without home high-speed internet were also provided with a Hotspot in order to ensure connectivity. If necessary, participants were taught how to use the iPad and Zoom application by a member of the study team prior to the first meeting and the study coordinator was on the call for every group meeting to assist with any technological difficulties.

The MBSR program was run by a board-certified psychologist experienced in MBSR administration. Treatment cohorts were assembled at the beginning of each month with patients recruited the month prior, resulting in groups varying in size from 5 to 15 participants. The course was modeled after a well-established protocol, first designed by Jon Kabat-Zinn (39), and used in multiple studies investigating the effect of MBSR on individuals with chronic disease (40). However, our sessions took place online, necessitating a slightly modified version (m-MBSR). In addition to being necessitated by the COVID-19 pandemic, prior research looking at efficacy of online mindfulness-based interventions (MBIs) provided further rationale (41). Participants met once a week for 1.5 h over Zoom, rather than the usual 2.5 h in person, and the silent retreat at week 7 was 3 h instead of the traditional 7. In this setting, the sessions were shortened intentionally due to concerns of zoom fatigue and length of attention, especially in this patient population, and due to research supporting the benefits of abbreviated MBSR (42, 43). In place of the full yoga routine typically administered in person, the modified version used a gentle, seated stretching to utilize similar calming body movements. This modification was in part due to the abbreviated sessions, as well as the fear of gait instability in this population. In addition to the weekly meeting, patients were provided with both formal and informal practices each week as homework, including guided meditation practices to listen to daily and ways to bring mindfulness to daily life as a part of the program.

To control for socialization as a confounding factor, an online Stroke Support Group (SSG) was used as the comparator intervention. This SSG program was run by the Stroke Program’s nurse practitioners and was also modified due to the pandemic, meeting once a week for 40 min over Zoom rather than once a month for 2 h in person. Consistent with a typical stroke support group, along with providing support and resources for stroke survivors and their families, topics pertaining to stroke recovery, the effect stroke has on patients and their caretakers, and steps patients can take to prioritize their health were discussed. Patients were encouraged to actively participate with questions and comments.

For additional comparison, an existing study population of 128 age-similar patients with minor stroke who did not undergo either intervention but had cognitive testing at similar time points was also included in the study as a control arm to evaluate the hypothesis that both m-MBSR and online SSG may be more effective than no intervention on improving patient reported outcomes and cognitive measures. These patients were part of our existing stroke registry and evaluated at 1- and 6-months post-stroke as part of routine clinical care. These patients were recruited utilizing the same inclusion and exclusion criteria as the MBSR and SSG groups.

### Engagement

Based on prior studies on MBSR evaluating participation in the practice and potential concern that individuals with cognitive impairment may have difficulty with focus and attention impairing their ability to be present, engagement was assessed over the course of the intervention. While prior studies classified engagement as physical attendance of the class, other studies have seen better results with more nuanced definitions, including homework completion and psychological engagement with the material (44). For this study, engagement was defined as the level of alertness and participation displayed by the participant throughout the class. Behaviors of each patient were observed by both the study coordinator (SG) and the intervention leader (NG for MBSR, DM or EL for SSG) during each session. These behaviors included: attention paid to the screen during presentations (eye contact with the speaker, nodding along to the discussion, following along with the PowerPoint presentations), ability to follow directions (actively performing the breathing techniques, movements, or session activities at the direction of the leader), participation in the discussion (asking questions, responding to the session leader’s prompts), whether they were competing unrelated tasks during the session (cooking, talking to family members, moving around the room and off screen frequently), and the amount of homework reported as complete each week (MBSR group only). Engagement was assessed and reported at the end of each session as a single dichotomous variable—“engaged” or “not engaged” rather than a scale of engagement, as individuals clearly fell into two unique groups. When it became evident that patients demonstrated the same level engagement throughout the entirety of the intervention, the variable was also collapsed into a single value representative of participation over the entire intervention period, for ease of analysis. Any disagreements between reviewers (*n* = 2) were resolved through consensus at the completion of the study.

### Clinical and cognitive testing

Patient-reported outcome metrics evaluating overall function, mood, and satisfaction were assessed pre- and post-intervention, along with cognitive measures. To evaluate patient-reported outcomes, participants were administered the Stroke Impact Scale (function), Patient Health Questionnaire (depression), Functional Assessment of Chronic Illness Therapy (FACIT) (fatigue), Barthel Index for Activities of Daily Living (function), and Patient Reported Outcomes Measurement Information System (PROMIS) measures (mood, fatigue, and ADLs) at each clinic visit.

A battery of cognitive tests, developed in conjunction with our neuropsychologist to be efficient yet sensitive to cognitive dysfunction commonly faced by those with minor stroke (10), was used to evaluate verbal and spatial memory, motor and processing speed, and executive function. Tests included: the Delis-Kaplan Executive Function System (D-KEFS) (45), Hopkins Verbal Learning Test (44), Brief Visuospatial Memory Test–Revised (46), Symbol Digit Modalities Test (47), and Grooved Pegboard Test (48). The Montreal Cognitive Assessment (MoCA) (13) was also included as a brief screen of global cognition. T-scores were used to describe normative data. If a given task was traditionally described using a z-score (cutoff −4 to 4), it was converted to a *T*-score for consistency and to allow averaging of scores across tasks. Additional information, including patient demographics (age, sex, self-identified race, and education), stroke characteristics (admission and discharge NIHSS (49) score, infarcted hemisphere, lesion volume, and cortical vs. subcortical location), social support (living with someone at home), functional baseline (pre-stroke mRS score), and medical comorbidities (history of smoking, hypertension, diabetes, depression, and Charlson Comorbidity Index), was also collected.

### Cognitive analysis

For each cognitive test, *T*-scores were calculated from raw scores using the age-specific normative data according to the corresponding test manual. Cognitive tests were then divided into 6 different domains. Within each domain, *T-*scores were averaged to generate a composite domain score: *Verbal Memory*—Hopkins Verbal Learning Test total learning and Hopkins Verbal Learning Test delayed recall; *Motor Processing Speed*—D-KEFS Trail Making trial 5, Grooved Pegboard Test dominant hand, and Grooved Pegboard Test nondominant hand; *Spatial Memory*—Brief Visuospatial Memory Test–Revised total learning and Brief Visuospatial Memory Test–Revised delayed recall; *Processing Speed*—Symbol Digit Modalities Test written trial, Symbol Digit Modalities Test oral trial, and D-KEFS Trail Making trial 1; *Executive Function*—D-KEFS letter fluency, D-KEFS category fluency, and D-KEFS Trails Making trials 2, 3, and 4; and *Overall Global Function*.

### Statistical analysis

Data were analyzed using Stata version 14 (College Station, TX, USA). To determine the impact of m-MBSR on post-stroke cognitive impairment, the primary aim of this study, ANOVA was used to compare performance on PROs and cognitive tasks between all three groups at each time point, as well as averaged cognitive domains. Changes in score over time were also compared. Based on a sample size of 15 patients per group, with respect to reported *T* scores we anticipated 80% power to detect a difference in mean performance of 10.5 points, or approximately one standard deviation, between groups.

To assess the impact of engagement on outcome, independent t-tests were used within the m-MBSR cohort to compare performance for those engaged versus not engaged.

## Results

Thirty patients were randomized to Stroke Support Group (*n* = 14, 7 male (50%)) or modified-Mindfulness Based Stress Reduction (*n* = 16, 9 male (56%)). Repeat evaluation was performed following the 8-week intervention. By the second clinic visit, one patient had dropped out of the study and a second was censored due to recurrent stroke resulting in post-intervention clinical data from 28 patients (SSG *n* = 14, m-MBSR *n* = 14). Seventy-nine (62%) of the 128 patients in the control group who were evaluated at 1 month but did not undergo intervention returned for follow-up testing. Their follow-up was somewhat later than the other two groups, on average 6.8 (compared to 4.1) months post-stroke.

### Group comparisons—demographics and stroke severity

The average age of the entire cohort was 64.7 years. Forty-nine percent was male; 27% black. The three groups were similar in age, race, and sex. Medical comorbidities, measured by the Charleston Comorbidity Index (CCI) and premorbid mRS scores were consistent across intervention groups (SSG: 1.8 and 0.31 respectively; m-MBSR: 1.4 and 0.31); though pre-stroke mRS was significantly lower for the much larger control group (0.06, *p* = 0.004) (see Table 1 for full details). Stroke severity was comparable across all groups, with a mean admission and discharge NIHSS for the entire cohort of 2.7 and 1.6, respectively. The infarct volumes were small, ranging from 0.06 to 18.9 cc, with greater than 85% of recruited patients having an infarct size under 5 cc. Ten participants (71.4%) in SSG and 10 in Mindfulness (62.5%) had infarcts within the left hemisphere – a slightly higher proportion than controls (*n* = 60 (41.2%)). While the percentage of patients with isolated subcortical strokes was not significantly different across groups (SSG: 8 (57.1%), m-MBSR: 10 (62.5%), control: 74 (59.2%), *p* = 0.953), the percentage of patients with isolated cortical locations did differ (SSG: 6 (42.9%), m-MBSR: 6 (37.5%), control: 19 (15.2%), *p* = 0.009).

**Table 1:** Patient characteristics.

Variable	Total	Stroke support group	Mindfulness group	Control group	*p*-values
	(*N* = 158)	(*N* = 14)	(*N* = 16)	(*N* = 128)	
Demographics					
Age, *mean years (SD)*	64.7 (13.6)	63.6 (17.2)	67.9 (10.7)	64.4 (13.5)	0.603
Sex, *n male (%)*	77 (48.7%)	7 (50.0%)	9 (56.3%)	61 (47.7%)	0.806
Race, *n black (%)*	43 (27.2%)	5 (35.7%)	4 (25.0%)	34 (26.6%)	**0.050**
Education, *mean years (SD)*	13.5 (2.6)	14.6 (3.2)	12.8 (1.8)	13.4 (2.6)	0.153
Premorbid IQ, *mean (SD)*	107.1 (11.4)	110.9 (13.6)	105.4 (6.5)	106.8 (11.5)	0.442
Handedness, *n right (%)*	129 (87.2%)	13 (100.0%)	12 (80.0%)	104 (86.7%)	0.571
Returned to work, *n yes (%)*	29 (28.2%)	3 (25.0%)	6 (40.0%)	20 (26.3%)	0.542
Social support, *n yes (%)*	140 (89.7%)	12 (85.7%)	13 (81.3%)	115 (91.3%)	0.403
Occupation code, *n (%)*					0.543
1. Professional	18 (14.1%)	4 (33.3%)	0 (0.0%)	14 (13.3%)	
2. Intermediate	23 (18.0%)	2 (16.7%)	4 (36.4%)	17 (16.2%)	
3. Skilled	28 (21.9%)	2 (16.7%)	2 (18.2%)	24 (22.9%)	
4. Semiskilled	38 (29.7%)	2 (16.7%)	4 (36.4%)	32 (30.5%)	
5. Unskilled	19 (14.8%)	2 (16.7%)	1 (9.1%)	16 (15.2%)	
Medical history					
Charleston comorbidity index, *mean (SD)*	1.8 (1.4)	1.8 (2.5)	1.4 (1.0)	1.9 (1.4)	0.554
Prestroke mRS, *mean (SD)*	0.11 (0.37)	0.31 (0.63)	0.31 (0.60)	0.06 (0.27)	**0.004**
Prior stroke, *n yes (%)*	25 (15.9%)	1 (7.1%)	4 (25.0%)	20 (15.8%)	0.408
Depression, *n yes (%)*	25 (16.2%)	3 (21.4%)	3 (18.8%)	19 (15.3%)	0.957
Smoking, *n yes (%)*	44 (28.0%)	7 (50.0%)	5 (31.3%)	32 (25.2%)	0.140
Hypertension, *n yes (%)*	121 (77.6%)	12 (85.7%)	9 (56.3%)	100 (79.4%)	0.084
Diabetes, *n yes (%)*	59 (37.8%)	6 (42.9%)	6 (37.5%)	47 (37.3%)	0.920
Hyperlipidemia, *n yes (%)*	106 (68.0%)	8 (57.1%)	7 (43.8%)	91 (72.2%)	**0.047**
Driving, *n yes (%)*	67 (47.9%)	4 (33.3%)	6 (40.0%)	57 (50.4%)	0.430
Stroke characteristics					
Admission NIHSS, *mean points (SD)*	2.70 (2.34)	2.08 (2.10)	2.63 (1.45)	2.78 (2.46)	0.587
Stroke volume, *mean cc (SD)*	4.68 (10.5)	2.0 (5.1)	0.8 (0.9)	5.4 (11.4)	0.187
Hemisphere, *n left (%)*	80 (51.0%)	10 (71.4%)	10 (62.5%)	60 (47.2%)	0.142
Isolated subcortical location, *n yes (%)*	92 (59.4%)	8 (57.1%)	10 (62.5%)	74 (59.2%)	0.953
Isolated cortical location, *n yes (%)*	31 (20.0%)	6 (42.9%)	6 (37.5%)	19 (15.2%)	**0.009**
Discharge NIHSS, *mean points (SD)*	1.59 (1.97)	1.0 (1.2)	1.7 (1.7)	1.6 (2.1)	0.548
White matter disease score—CHS, *n (%)*					0.655
1	17 (11.7%)	2 (15.4%)	4 (28.6%)	11 (9.3%)	
2	62 (42.8%)	6 (46.2%)	5 (35.7%)	51 (43.2%)	
3	33 (22.8%)	4 (30.8%)	1 (7.1%)	28 (23.7%)	
4	18 (12.4%)	1 (7.7%)	3 (21.4%)	14 (11.9%)	
5	10 (6.9%)	0 (0.0%)	1 (7.1%)	9 (7.6%)	
6	3 (2.1%)	0 (0.0%)	0 (0.0%)	3 (2.5%)	
TOAST, *n (%)*					0.597
Large vessel	42 (27.3%)	3 (25.0%)	4 (26.7%)	35 (27.6%)	
Cardioembolism	22 (14.3%)	1 (8.3%)	1 (6.7%)	20 (15.8%)	
Small vessel	66 (42.9%)	5 (41.7%)	6 (40.0%)	55 (43.3%)	
Other determined etiology	8 (5.2%)	0 (0.0%)	1 (6.7%)	7 (5.5%)	
Undetermined etiology	16 (10.4%)	3 (25.0%)	3 (20.0%)	10 (7.9%)	
One month mRS, *n (%)*					0.455
0	45 (28.9%)	5 (35.7%)	2 (12.5%)	38 (30.2%)	
1	70 (44.9%)	4 (28.6%)	11 (68.8%)	55 (43.7%)	
2	27 (17.3%)	3 (21.4%)	1 (6.3%)	23 (18.3%)	
3	8 (5.1%)	2 (14.3%)	1 (6.3%)	5 (4.0%)	
4	5 (3.2%)	0 (0.0%)	1 (6.3%)	4 (3.2%)	
5	1 (0.6%)	0 (0.0%)	0 (0.0%)	1 (0.8%)	
Rehab, *n (%)*					**0.005**
None	44 (30.8%)	8 (57.1%)	7 (46.7%)	29 (25.4%)	
Inpatient	29 (20.3%)	2 (14.3%)	3 (20.0%)	24 (21.1%)	
Home	32 (22.4%)	1 (7.1%)	1 (6.7%)	30 (26.3%)	
Outpatient	35 (24.5%)	2 (14.3%)	3 (20.0%)	30 (26.3%)	
Inpatient and Home	3 (2.1%)	1 (7.1%)	1 (6.7%)	1 (0.9%)	

*P*-values of of statistical significance (< 0.05) are bolded.

### Group comparisons—functional assessment and patient reported outcomes

Group comparisons of cognitive function and PROs are detailed in Table 2. Functional outcomes at the baseline visit were similar across groups. Those randomized to online SSG had an average NIHSS of 1.0 and mRS of 1.1, compared to an NIHSS of 1.6 and mRS of 1.3 in the m-MBSR group (*p* = ns). All groups saw improvement by their post-intervention visit, as documented in Table 1.

**Table 2:** Group performance.

Variable	Baseline				Follow-up			
	Stroke support group (*N* = 14)	Mindfulness group (*N* = 16)	Control group (*N* = 128)	*p*-value	Stroke support group (*N* = 14)	Mindfulness group (*N* = 14)	Control group (*N* = 79)	*p*-value
Stroke characteristics, *mean (SD)*								
Barthel index (BI)	95.0 (10.2)	96.3 (10.4)	97.4 (8.7)	0.587	98.3 (5.8)	100.0 (0.0)	99.3 (3.2)	0.509
NIH stroke scale (NIHSS)	1.00 (1.57)	1.63 (1.89)	0.82 (1.48)	0.141	0.58 (1.00)	0.40 (0.52)	0.45 (1.23)	0.917
Modified Rankin scale (mRS)	1.14 (1.10)	1.25 (1.00)	1.09 (1.03)	0.831	1.17 (1.27)	0.90 (0.74)	0.72 (0.90)	0.299
Patient-reported outcomes, *mean (SD)*								
FACIT	36.4 (8.7)	32.0 (14.9)	37.3 (11.7)	0.374	37.6 (10.4)	30.7 (13.0)	38.2 (11.2)	0.164
PHQ-9	5.2 (5.9)	6.7 (7.3)	4.9 (5.8)	0.655	4.9 (3.9)	6.1 (6.9)	4.4 (5.2)	0.670
Likert-symptoms	6.0 (1.3)	6.2 (1.2)	5.5 (1.6)	0.312	5.4 (1.3)	5.3 (1.9)	5.7 (1.5)	0.771
Likert-QOL	6.1 (1.1)	5.7 (2.2)	5.2 (1.9)	0.349	5.1 (1.8)	5.2 (1.9)	5.5 (1.6)	0.779
Percent recovered	79.8 (18.2)	76.8 (24.1)	76.3 (23.3)	0.918	76.4 (19.3)	77.5 (19.3)	80.6 (17.6)	0.778
Stroke impact scale								
Strength	90.8 (11.1)	72.5 (23.1)	79.1 (22.1)	0.294	76.7 (22.5)	71.7 (24.8)	80.2 (20.2)	0.618
Memory and thinking	86.2 (13.4)	77.1 (19.6)	87.9 (16.7)	0.224	75.7 (12.9)	84.3 (11.5)	88.1 (12.9)	0.086
Mood	86.6 (11.7)	78.5 (9.0)	83.9 (17.2)	0.583	76.4 (9.8)	74.2 (22.5)	83.9 (13.8)	0.233
Communication	96.2 (6.9)	89.2 (13.7)	92.3 (15.8)	0.687	81.1 (20.2)	90.2 (15.9)	93.5 (10.9)	0.112
Activities of daily living	98.7 (1.6)	85.0 (22.0)	89.2 (18.8)	0.383	78.0 (28.3)	86.0 (15.2)	95.6 (12.2)	**0.025**
Mobility	96.3 (5.4)	89.2 (19.6)	85.0 (19.3)	0.337	77.8 (20.7)	81.8 (19.7)	90.7 (13.1)	0.102
Fine motor movements	96.0 (8.0)	91.4 (21.0)	84.2 (23.4)	0.367	68.8 (35.6)	83.0 (21.8)	91.6 (12.6)	0.015
Socialization	92.5 (6.9)	86.8 (27.4)	78.5 (21.5)	0.217	85.6 (10.1)	75.5 (33.2)	86.7 (17.0)	0.450
PROMIS measures								
Anxiety (lower better)	8.6 (0.9)	6.3 (0.9)		0.092	7.3 (1.1)	7.1 (0.9)		0.868
Depression (lower better)	6.4 (0.9)	6.1 (1.1)		0.872	5.7 (0.8)	5.9 (0.9)		0.841
Fatigue (lower better)	9.5 (1.0)	10.6 (1.3)		0.531	7.6 (0.8)	9.9 (1.1)		0.114
Sleep (lower better)	10.4 (1.1)	9.1 (1.0)		0.351	10.5 (0.9)	9.0 (1.1)		0.302
Social (higher better)	14.6 (1.4)	16.8 (1.2)		0.259	17.1 (1.2)	15.3 (1.2)		0.302
Pain (lower better)	6.9 (1.2)	7.8 (1.4)		0.638	5.8 (0.9)	9.0 (1.6)		0.103
Intensity (lower better)	2.1 (0.8)	2.6 (0.8)		0.652	1.8 (0.8)	4.0 (0.9)		0.092
Physical (higher better)	16.1 (1.4)	15.9 (1.2)		0.887	17.3 (1.1)	15.6 (1.3)		0.350
Anxiety 8a (lower better)	15.7 (1.6)	13.5 (1.9)		0.377	15.3 (1.8)	14.7 (2.2)		0.835
Depression 8a (lower better)	13.1 (1.7)	12.3 (1.6)		0.725	11.2 (1.3)	12.4 (1.8)		0.594
Self efficacy 4a (higher better)	14.9 (1.4)	16.2 (0.9)		0.446	15.5 (1.0)	14.5 (1.5)		0.579
Cognitive testing *T* scores, *mean (SD)*								
MoCA	22.5 (5.9)	23.5 (5.2)	24.3 (3.7)	0.231	23.7 (6.0)	25.5 (3.7)	25.5 (3.4)	0.276
Peg board task (TIMES)								
Dominant hand	23.7 (67.1)	14.8 (61.2)	22.1 (65.8)	0.595	21.7 (67.4)	21.9 (66.5)	27.5 (71.0)	0.578
Nondominant hand	25.8 (68.5)	20.6 (66.9)	22.1 (66.1)	0.433	23.1 (65.9)	22.3 (67.7)	26.8 (67.9)	0.979
DKEFS letter fluency task								
Letter fluency	43.1 (16.0)	42.3 (14.4)	46.9 (13.8)	0.345	44.7 (14.4)	50.4 (13.7)	49.5 (13.4)	0.445
DKEFS category fluency task								
Category fluency	39.9 (13.1)	43.8 (10.3)	44.7 (13.0)	0.401	44.4 (16.2)	46.6 (9.8)	46.8 (13.3)	0.820
DKEFS category switching task								
Category switching	43.6 (17.0)	45.6 (9.1)	45.4 (13.1)	0.889	52.4 (14.9)	51.2 (13.8)	47.4 (12.3)	0.287
Switching accuracy	45.7 (16.0)	48.8 (8.6)	46.1 (12.0)	0.684	53.1 (13.7)	50.2 (13.3)	47.5 (11.8)	0.250
DKEFS trails task								
Trail 1	33.8 (14.3)	35.4 (14.1)	30.6 (13.5)	0.330	36.6 (16.6)	40.1 (12.2)	44.7 (13.6)	0.097
Trail 2	38.8 (13.2)	43.5 (16.2)	41.0 (13.3)	0.654	36.3 (14.4)	44.8 (12.6)	45.5 (13.2)	0.061
Trail 3	36.0 (12.7)	39.8 (15.7)	41.5 (13.2)	0.352	39.1 (13.8)	43.1 (14.9)	46.1 (11.5)	0.132
Trail 4	42.7 (11.8)	41.6 (14.6)	47.0 (13.5)	0.217	42.6 (15.1)	49.6 (13.5)	48.2 (13.0)	0.323
Trail 5	33.7 (12.4)	43.1 (9.6)	44.3 (11.5)	**0.008**	34.3 (14.2)	41.3 (13.5)	45.8 (11.2)	**0.004**
HVLT task								
Total recall	32.9 (7.7)	33.1 (16.4)	30.5 (9.0)	0.460	36.9 (12.5)	32.0 (12.0)	33.5 (9.6)	0.431
Delayed recall	33.9 (11.8)	30.8 (11.7)	30.0 (10.1)	0.414	34.9 (13.4)	32.6 (11.0)	34.0 (11.1)	0.871
Recognition	40.7 (14.1)	35.1 (10.6)	33.3 (12.9)	0.119	37.6 (14.5)	38.0 (10.5)	35.7 (11.8)	0.734
Retention	38.4 (14.0)	37.5 (19.9)	35.6 (14.6)	0.748	36.4 (14.3)	36.9 (14.9)	39.1 (14.3)	0.732
BVMT task								
Total recall	34.3 (12.6)	44.1 (14.2)	41.6 (12.6)	0.081	40.2 (15.1)	42.8 (16.2)	43.7 (14.3)	0.712
Delayed recall	39.1 (13.1)	46.9 (13.9)	42.7 (13.8)	0.312	37.6 (16.5)	46.3 (17.6)	44.7 (15.1)	0.256
SDMT								
Written	41.4 (14.3)	37.6 (11.3)	41.5 (12.7)	0.508	42.0 (13.2)	43.8 (13.2)	45.0 (12.1)	0.697
Oral	40.5 (12.4)	40.1 (12.7)	37.9 (11.9)	0.623	42.6 (14.1)	42.9 (12.4)	39.9 (11.5)	0.572

*P*-values of of statistical significance (< 0.05) are bolded.

Baseline Patient Reported Outcomes (PROs) were also similar across groups. While the modified-MBSR group displayed slightly higher levels of depression on the PHQ-9 [6.7 versus 5.2 (*p* = ns)], the online SSG reported higher anxiety levels [8.6 versus 6.3 (*p* = ns)] on the PROMIS anxiety section. Individuals in the SSG reported slightly higher scores on the Stroke Impact Scale for every category, indicating higher mood, better mobility, more socialization and ADLs, and feeling less cognitively slow although results were not statistically significant. Interestingly, by the second visit, the higher Stroke Impact Scale scores for 5 of the 8 categories switched to the m-MBSR group, though remained non-significant. The control group did report significantly better scores for the Activities of Daily Living (ADLs) and Fine Motor categories compared to those in the SSG at post-intervention follow-up, which may have been secondary to the extended period of time allowed for recovery. However, there were no other major differences.

### Group comparisons—cognitive testing

Cognitive testing scores were also similar at the baseline time point between groups, with average (SD) MoCA scores of 22.5 (5.9), 23.5 (5.2) and 24.3 (2.7) for the SSG, m-MBSR, and control groups, respectively. While overall scores remained similar at the second visit, the m-MBSR average (SD) MoCA score of 25.5 (3.7) appeared more consistent with the control group’s 25.5 (3.4) than the SSG, with an average of only 23.7 (6.0). As expected, cognitive scores improved consistently for all groups between the first and second visits, though statistically insignificant. The SSG group demonstrated a slower D-KEFS Trails Task 5 score at baseline (*T* = 33.7 (12.4)), almost one standard deviation below the m-MBSR group and a full SD below the control group (*T* = 43.1 (9.6) and *T* = 44.3 (11.5) respectively, *p* = 0.008). This difference persisted at follow-up (*p* = 0.004). There were no other significant differences in performance across groups.

### Changes in scores post-intervention

**Figure 1:**
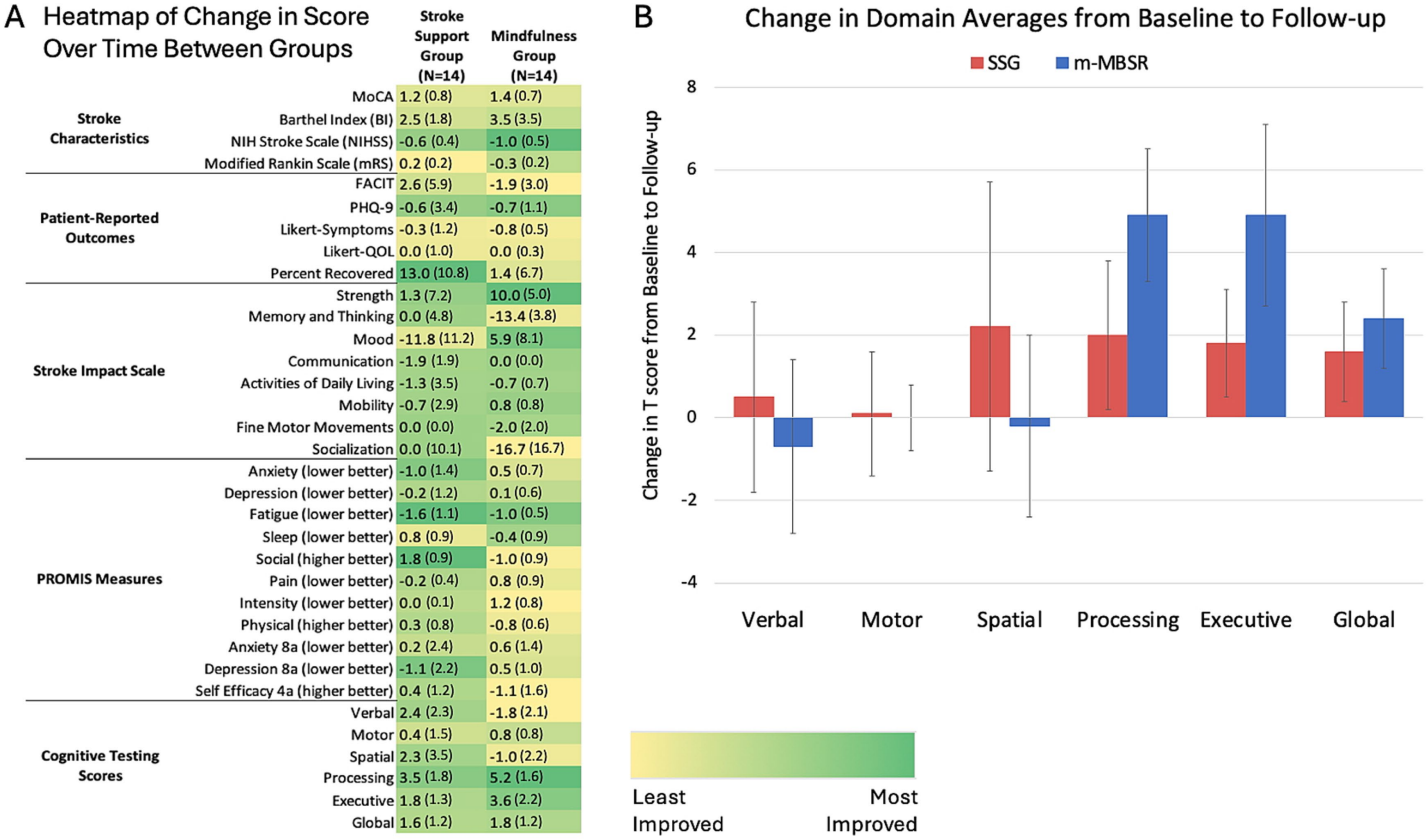
Group differences. **(A)** Patients in the m-MBSR group demonstrated a greater degree of improvement pre- to post-intervention for objectively measured outcomes of performance following intervention, while the SSG tended to show more improvement on patient-reported metrics. Results are displayed as mean change (and standard error) in raw score, or *T* score for cognitive domains. A greater intensity of green shading corresponds to a larger improvement in score over time and allows for comparison of improvement across groups, regardless of whether a positive or negative value represents improvement. **(B)** Specifically, patients in the m-MBSR group demonstrated more improvement than SSG in processing speed, executive function, and global processing domains, though not statistically significant. Average changes in *T* score pre- to post-intervention for each cognitive domain are shown for each group, along with the standard error represented by error bars for each value. Ten points represents one standard deviation on the *T* scale.

Given that all groups were expected to recover and that differences were only modest between intervention groups at both time points and statistically insignificant, a heat map was generated to better visualize any pattern of differences in improvement over time between groups (Figure 1). Those participating in m-MBSR showed greater degrees of improvement in objective functional measures, such as the BI and mRS, and patient reported assessments of function (SIS, Likert scales perception of recovery), while the SSG showed greater degrees of improvement on the patient-reported PROMIS measures evaluating emotional wellbeing. The SSG also improved to a greater extent in the areas of verbal and spatial memory, while m-MBSR participants demonstrated more improvement in processing speed, executive function, and global cognition.

### Engagement

Nine individuals (60%) within the m-MBSR group were actively engaged throughout the duration of the intervention, while 6 were not. Eleven of 14 were engaged during the SSG (78.6%). Within the m-MBSR group, we saw no significant differences in cognitive performance between the engaged and unengaged groups (Table 3). However, it was notable that the engaged group tended to perform better both pre- and post-intervention compared to the unengaged group.

**Table 3:** Effect of engagement.

Variable	Baseline			Follow-up		
	Unengaged (*n* = 6)	Engaged (*n* = 9)	*p*-value	Unengaged (*n* = 5)	Engaged (*n* = 9)	*p*-value
Stroke characteristics, *mean (SD)*						
MoCA	21.3 (2.2)	25.1 (1.7)	0.192	24.4 (2.3)	26.1 (0.9)	0.428
Barthel index (BI)	95.8 (4.2)	96.1 (3.9)	0.963	100 (0.0)	100 (0.0)	1.000
NIH stroke scale (NIHSS)	1.67 (1.09)	1.67 (0.50)	1.000	0.2 (0.2)	0.6 (0.2)	0.242
Modified Rankin scale (mRS)	1.17 (0.40)	1.33 (0.37)	0.772	0.6 (0.2)	1.2 (0.4)	0.217
Patient-reported outcomes, *mean (SD)*						
FACIT	34.8 (7.1)	28.6 (5.6)	0.519	31.5 (6.9)	30.2 (5.6)	0.884
PHQ-9	6.2 (4.2)	7.2 (2.4)	0.842	8.5 (4.8)	4.5 (2.0)	0.401
Likert-symptoms	6.5 (0.5)	6.0 (0.6)	0.571	5.0 (1.2)	5.5 (0.8)	0.732
Likert-QOL	5.6 (0.9)	5.8 (1.2)	0.896	5.0 (1.0)	5.3 (0.8)	0.818
Percent recovered	72.0 (11.6)	80.8 (9.9)	0.573	85.0 (2.9)	72.5 (9.8)	0.346
Stroke impact scale						
Strength	80.0 (10.8)	65.0 (12.6)	0.401	77.5 (13.6)	60.0 (15.0)	0.478
Memory and thinking	85.7 (9.5)	68.6 (9.3)	0.243	83.5 (7.0)	85.7 (5.7)	0.855
Mood	76.1 (5.2)	80.5 (3.7)	0.507	60.7 (10.4)	94.4 (3.3)	0.090
Communication	88.6 (6.7)	89.7 (6.9)	0.910	83.4 (16.7)	97.1 (2.9)	0.500
Activities of daily living	87.5 (11.8)	82.5 (11.7)	0.774	90.0 (10.0)	80.0 (10.0)	0.550
Mobility	99.3 (0.7)	81.7 (12.1)	0.275	87.4 (12.6)	73.4 (13.4)	0.515
Fine motor movements	100.0 (0.0)	85.0 (13.7)	0.397	76.0 (24.0)	90.0 (6.0)	0.629
Socialization	98.3 (1.7)	78.1 (17.7)	0.381	60.0 (20.8)	98.8 (1.3)	0.245
PROMIS measures						
Anxiety (lower better)	7.5 (2.3)	5.7 (0.7)	0.391	8.6 (2.3)	6.2 (0.7)	0.240
Depression (lower better)	8.5 (2.6)	4.8 (0.4)	0.108	6.8 (2.3)	5.4 (0.6)	0.479
Fatigue (lower better)	8.7 (2.4)	12.3 (1.6)	0.203	8.2 (2.2)	10.9 (1.2)	0.265
Sleep (lower better)	10.7 (2.0)	8.4 (1.0)	0.292	9.0 (2.4)	9.0 (1.3)	1.000
Social (higher better)	18.3 (1.7)	15.3 (1.8)	0.269	16.8 (2.2)	14.4 (1.5)	0.378
Pain (lower better)	6.2 (2.0)	9.2 (2.1)	0.327	7.2 (3.2)	10 (1.8)	0.427
Intensity (lower better)	1.9 (1.4)	3.3 (1.0)	0.421	3.7 (1.9)	4.1 (1.1)	0.840
Physical (higher better)	15.3 (2.5)	15.9 (1.6)	0.845	17.8 (1.6)	14.4 (1.8)	0.233
Anxiety 8a (lower better)	16.5 (4.6)	12.0 (1.2)	0.278	15.6 (5.3)	14.2 (2.0)	0.775
Depression 8a (lower better)	15.5 (4.0)	10.7 (0.6)	0.161	13.6 (4.7)	11.8 (1.3)	0.638
Self efficacy 4a (higher better)	16.3 (1.6)	15.7 (1.3)	0.746	11.0 (3.5)	16.4 (1.0)	0.086
Cognitive domains, *mean (SD)*						
Verbal	24.5 (1.8)	39.1 (4.3)	**0.031**	24.0 (2.6)	36.9 (3.0)	**0.013**
Motor	32.6 (3.6)	31.6 (2.9)	0.831	33.2 (4.3)	31.5 (2.3)	0.701
Spatial	47.8 (5.8)	42.8 (5.1)	0.534	50.1 (7.7)	41.4 (5.4)	0.367
Processing	31.8 (2.6)	40.2 (4.0)	0.146	40.6 (4.9)	43.2 (4.0)	0.693
Executive	40.9 (3.4)	43.6 (4.8)	0.677	45.0 (4.6)	48.0 (4.1)	0.648
Global	36.3 (2.6)	39.9 (3.2)	0.437	39.6 (3.3)	41.4 (3.0)	0.720

*P*-values of of statistical significance (< 0.05) are bolded.

## Discussion

Our results suggest that while differences were at best modest, modified-Mindfulness Based Stress Reduction (m-MBSR) may have some benefit on cognitive function during the early stages of recovery. Though all groups recovered from their baseline visit to follow-up post-intervention without significant differences between them, those undergoing m-MBSR appeared more likely to improve to a greater extent in more cognitive domains, especially those involving frontal lobes including processing speed and executive function. Interestingly, those in the SSG showed more improvement in their self-reported PROMIS metrics. Importantly, engagement did not necessarily correlate with better results; however, only 2/3 of the participants were able to visibly and actively engage at this point in their recovery.

M-MBSR is a practice thought to alter the brain by engaging the frontal lobes. A meta-analysis of 21 neuroimaging studies in 2014 found that the pre-frontal cortex, anterior cingulate, and orbitofrontal cortex were all involved during mindful meditation, along with the sensory cortex, the insula, and hippocampal regions (18). A study of Tibetan monks also showed changes in pre-frontal delta, beta, and gamma activity during deep meditation, with corresponding shifts in proteomics revealing sources located within the attention and emotion networks (17). It follows with the hypothesis that activities highly dependent on the frontal lobes, including mood regulation and cognitive tasks dependent on networks involving these regions, may benefit from these changes. Previous studies involving patients with chronic health conditions provide supporting evidence (19–31). However, m-MBSR has not been substantially studied in patients following stroke, and particularly those with minor stroke in the early stages of recovery. In this population, we have demonstrated previously that a lesion in any location can result in difficulties with processing speed and executive function (10), and that areas including the bilateral frontoparietal cortices show atypical beta activity (38), serving as a potential target for behavioral intervention with MBSR. Clinically, following a course of m-MBSR there does appear to be some benefit, particularly with respect to tasks such as Symbol Digit Modalities written symbol task, and D-KEFS-Trail Making #4, which rely heavily on frontal lobe regions. Studies involving chronic stroke patients showed improvement on these tasks to an even greater degree (31). Imaging correlates will need to be further studied.

While cognitive testing results demonstrated small but expected patterns—with patients demonstrating better attention and executive function after m-MBSR compared to those participating in SSG—results for patient-reported outcomes were more surprising. Though both groups had similar PROMIS scores at baseline, SSG patients reported greater improvements in many of the emotional markers following intervention compared to the m-MBSR group. This appears to contradict prior studies of m-MBSR; however, one explanation is that mindfulness involves body awareness and encourages patients to be more in tune with their symptoms. It is possible that following stroke this may have resulted in an increased sensitivity to post-stroke changes and, therefore, increased recognition of the effects of their deficits on their bodies and quality of life. Alternatively, it is possible that the SSG provided greater group cohesion and support for undergoing a stroke through thorough group discussion addressing stroke-specific concerns—compared to m-MBSR’s focus on more tools-based learning and meditation—which might result in inflated subjective reporting of recovery for SSG. Studies have also been conducted on the potential harms of MBSR practices and related practices like meditation and therapy, which suggest potential drawbacks of these interventions including more pronounced emotional difficulties (50–53); though whether these would outweigh the other benefits of MBSR remains unclear.

While m-MBSR did appear to result in small differences in cognition, they were not as significant as expected. This may be due to several key factors. Groups where brain changes have been observed following mindful meditation, such as Tibetan monks or much younger patient populations, are typically experienced with the practice and able to actively engage (17, 54). It would be rational to assume that some level of engagement is required to achieve both clinical and radiographic effects. While patients with minor stroke have low stroke severity overall, there is significant cognitive dysfunction to keep many from returning to work in the subacute setting, potentially posing a challenge to active practice. As the cognitive results did not differ significantly between the intervention groups, evaluating the impact of engagement was necessary. Results did not suggest that level of engagement was a significant predictor of clinical outcome. However, it is critical to point out that when evaluating participants’ levels of involvement in class participation, home practices, and other factors, we found less than 2/3 of participants to be actively engaged in the sessions, and engaged participants appeared, in general, to have better scores at baseline that persisted post-intervention. While the reasoning for the lack of engagement could be simple disinterest in the study, given that individuals voluntarily opted into these sessions, the lack of engagement may indicate that some patients with minor stroke are unable to engage to the level necessary to achieve benefit in the early stages of recovery. As the cognitive impairment within our cohort was mild and consistent given the low stroke severity of our population, we would not have expected to see differing levels of engagement based on cognitive deficits alone and confirmed that level of dysfunction, as assessed by performance on the MoCA pre-intervention, was not significantly related to subsequent level of “engagement.”

A second possibility for the modest clinical difference lies in the natural history of stroke recovery. As with language and motor deficits, a large majority of cognitive symptoms improve during the initial 6 months post-infarct (37). This makes implementing an intervention during this time attractive to optimize improvement. However, it can also make finding larger differences between groups more difficult, especially when deficits are already less severe at baseline for minor strokes. A more robust difference in results between groups may be expected during the chronic stage of recovery when deficits are more stable. Fortunately, even a small change in the degree of symptoms can make a large difference in a patient’s functional ability following minor stroke. Due to a lack of aphasia or hemiparesis, cognitive impairment is the predominant symptom for many minor stroke survivors, evidenced by scores on the MoCA that are several points lower than expected for age (13), resulting in difficulty returning to work (11). Conversely, the same small increase in MoCA, as seen following m-MBSR, could significantly improve function.

### Limitations

This study has several limitations. The relatively small cohort of patients with minor stroke was recruited from a single stroke center. In addition, baseline cognitive function was unknown. To account for this, *T*-scores were used to allow for comparison with the general population. Although it is possible that for some individuals a *T*-score <50 was normal, for the entire cohort to have such a poor baseline level given the high average years of education would be unlikely. Additionally, given the multiple administrations of cognitive assessments, it is also possible that some degree of observed improvement may have been due to practice effect. However, alternative versions of tests were used for subsequent evaluation when possible, and tests such as the MoCA have demonstrated reliability even when administered as frequently as every 3 months (55). It is important to note that our control group follow-up was later than the intervention groups, meaning they are 2–3 months farther into their recovery by the second visit. Interestingly, they were not much better than the intervention groups, suggesting the interventions may hasten recovery compared to no intervention. This will need to be investigated in future studies. There was also a higher rate of attrition for the control group (61.2% follow-up) compared to the SSG (100% follow-up) and MBSR (87.5% follow-up) groups; however, we compared the demographics for those who returned and those lost to follow-up and did not find significant differences. Remote intervention due to the Covid19 pandemic also poses its own unique set of challenges, including the lack of literature on designing an online MBSR course and possible internet connectivity issues, especially with an older population. The accessibility of taking the class in the comfort of their own home has allowed for greater participation and retention; however, home intervention also includes a greater potential for patients to be distracted and have issues utilizing zoom, potentially missing part of the intervention. Collecting a dichotomous engagement variable also posed challenges, as it is difficult to differentiate whether a patient was unable to engage due to their stroke or due to individual differences in personality and function. In addition, a more rigorous evaluation of the degree of engagement (e.g., amount of homework completed and patient perception of intervention) would provide greater insight into their level of engagement while at home as well as overall and how this relates to the effects of MBSR. Future studies are required to compare the effectiveness of the online version of m-MBSR to the in-person program, as well as to optimally assess the impact of engagement on treatment effect. Current results set the groundwork to continue to find beneficial interventions for post-stroke patients.

## Conclusion

Despite the aforementioned limitations, this study suggests that m-MBSR may provide a noninvasive, feasible therapeutic approach to improving cognitive deficits following minor stroke, specifically with respect to frontal lobe function. The modest effects seen in this study may be a consequence of m-MBSR requiring a level of engagement that many minor stroke patients in the acute setting are unable to achieve, and therefore m-MBSR may be more beneficial to improving cognition during the chronic stages of recovery; however, even modest effects have the potential to make a significant impact in this group. Future studies are needed to determine if intervention in the subacute setting results in the necessary changes in brain architecture, and whether using a biomarker such as functional neuroimaging to evaluate the integrity of cognitive networks allows for identification of individuals most likely to benefit at various stages post-stroke.

## Data Availability

The raw data supporting the conclusions of this article will be made available by the authors without undue reservation.
